# Necrology

**Published:** 1890-02

**Authors:** 


### NECROLOGY.

ALEXANDER LOCKHART, one of the best-known and most successful veterinary surgeons in this country, died at his home, 124 East Thirteenth Street, New York, on Friday evening, Jan. 9th, of rheumatic and heart troubles, aggravated by an attack of the influenza. He was born in Glasgow, Scotland, in 1842, and was educated at the Edinburgh Veterinary College and the Royal College of Veterinary Surgeons at London. He came to this city in 1866 and began business with his brother William, who had been here since 1849, and who was one of the most famous veterinary surgeons of his day.

In the last twenty years Mr. Lockhart had charge of the stables of Mr. Bonner, Mr. Belmont, A. T. Stewart, the Dwyer Brothers and many other noted horse owners, and he also looked after the stock of the Adams Express Company, the New York Cab Company and other large firms. He was for many years Professor of the Theory and Practice of Medicine at the New York College of Veterinary Surgeons.

